# CA19-9 as a Dynamic Biomarker for Continuous Monitoring of Therapeutic Efficacy in Pancreatic Adenocarcinoma

**DOI:** 10.3390/cancers17243902

**Published:** 2025-12-09

**Authors:** Luigi Brancato, Damar Osok, Laurent Van den Bossche, Eric Van Cutsem, Susan E. Bates, Johan Van den Bossche, Johannes Bogers

**Affiliations:** 1 ElmediX NV, Esperantolaan 4, 3001 Leuven, Belgium; dosok@kaviuon.org (D.O.); laurent.vandenbossche@elmedix.com (L.V.d.B.); johan.vandenbossche@elmedix.com (J.V.d.B.); 2 KAVI Institute of Clinical Research (KAVI-ICR), Faculty of Health Sciences, University of Nairobi, Nairobi P.O. Box 19676-00202, Kenya; 3 Laboratory of Cell Biology and Histology, Faculty of Medicine and Health Sciences, University of Antwerp, 2610 Antwerp, Belgium; 4 Department of Digestive Oncology, University Hospital Gasthuisberg and University of Leuven (KUL), 3000 Leuven, Belgium; eric.vancutsem@uzleuven.be; 5 Department of Medicine, Division of Hematology/Oncology, Columbia University Medical Center, New York, NY 10032, USA; seb2227@cumc.columbia.edu; 6 James J. Peters VA Medical Centre, Bronx, NY 10468, USA

**Keywords:** CA19-9, pancreatic adenocarcinoma, tumor marker, sensitivity and specificity

## Abstract

Pancreatic ductal adenocarcinoma (PDAC) remains one of the most lethal malignancies, largely due to late-stage diagnosis and limited therapeutic efficacy. The carbohydrate antigen 19-9 (CA19-9) is the most widely used serum biomarker in the management of PDAC. While CA19-9 has significant limitations as a screening or diagnostic tool, including low sensitivity for early-stage disease and a lack of expression in the Lewis antigen-negative population, its value in the post-diagnostic setting is well established. This review examines the production and clearance dynamics of CA19-9. It critically evaluates how these factors impact its role as a biomarker for prognosis, assessment of resectability, and real-time monitoring of therapeutic response and recurrence in patients with PDAC. We explore how the relatively short half-life and correlation with tumor burden make CA19-9 a dynamic tool for tracking disease progression and treatment efficacy, often providing insights that precede radiographic changes. This review concludes that, despite its limitations, CA19-9 remains an important, cost-effective, and widely accessible biomarker for the longitudinal management of patients with established pancreatic cancer. Its dynamic changes allow continuous real-time disease monitoring providing critical information for clinical decision-making.

## Simple Summary

Pancreatic ductal adenocarcinoma, one of the deadliest tumors, is often diagnosed too late for effective treatment. While limited as a tool for early detection, the blood biomarker CA19-9 excels in tracking tumor progression and monitoring treatment response. This review delves into the production and clearance mechanisms of CA19-9, revealing how its fast, dynamic changes can signal therapeutic effectiveness well before radiological imaging. Overshadowed by its known limitations as a diagnostic marker, CA19-9 remains underutilized in clinical practice. Yet, we believe this tumor biomarker can play a greater role in guiding treatment decisions.

## 1. Introduction

In 2022, it is estimated that globally there were 510,992 new cases of pancreatic cancer and 467,409 deaths making it the 12th most commonly diagnosed cancer and the 6th most common cause of cancer-related deaths [1]. Pancreatic ductal adenocarcinoma (PDAC) accounts for more than 90% of all pancreatic malignancies [2] and remains one of the most formidable challenges in modern oncology, characterized by a particularly aggressive biology, late-stage diagnosis, and profound resistance to conventional therapies [3,4]. The 5-year overall survival rate lingers at 12%, a figure that has seen only marginal improvement despite decades of research [5]. This grim outlook is primarily due to the aggressive biology of the tumor and the fact that most patients are diagnosed at an advanced, metastatic stage when curative treatment options are no longer available [6,7].

Given these challenges, it is widely accepted that the most impactful strategy to improve patient outcomes is to detect the disease at an earlier stage when curative-intent surgery is still possible [8,9,10]. The urgent need for novel, more accurate, and non-invasive biomarkers for the early detection of pancreatic cancer has spurred research efforts with a strong focus on liquid biopsies and exosomes, that could enable earlier and less invasive detection of pancreatic malignancy [10,11,12].

With ongoing efforts to improve early diagnosis, radiological imaging continues to play a key role in both the diagnosis and management of pancreatic cancer and is currently considered the gold standard for decision making by clinicians. The European Society of Medical Oncology (ESMO) guidelines recommend the use of computed tomography (CT) as the main modality for diagnosis; magnetic resonance imaging (MRI) is only recommended in the event that the CT is inconclusive [13]. CT is preferred based on its widespread availability and high diagnostic accuracy (sensitivity of 89–97%) [14]. Unless otherwise indicated, CT is also the imaging modality used for patient management. Imaging constitutes a major expense in cancer management [15]. Consequently, there is an urgent and unmet need for effective biomarkers that can aid in early detection, prognostication, and monitoring of treatment response [6]. Ideally, this would be a biomarker that is reliable relative to radiological imaging, cost-effective to allow for repeat tests at shorter intervals and readily accessible in the clinical setting.

Among the vast array of identified markers, Carbohydrate Antigen 19-9 (CA19-9) stands out as one of the most extensively studied and clinically utilized, in particular for PDAC [16,17]. First identified in 1979 from a human colorectal cancer cell line, CA19-9 is a carbohydrate antigen whose clinical utility was quickly recognized to be greatest in pancreatic adenocarcinoma [17]. For decades, it has remained the gold-standard serological marker for this devastating disease.

Studies indicate that, while CA19-9 is unsuitable for population screening, its levels correlate well with tumor burden and change rapidly in response to therapy, making it an invaluable tool for longitudinal patient management [18]. This review examines how the production and clearance dynamics of CA19-9 affect its validity and reliability as a tumor biomarker for assessing prognosis and therapeutic efficacy in pancreatic cancer. Most importantly, we will examine its overall performance as a monitoring biomarker for therapeutic efficacy compared to the gold standard, radiological imaging.

## 2. Production, Clearance, and Pathophysiological Role of CA19-9

The biochemical and biological mechanisms underlying CA19-9 production, secretion, and clearance are fundamental to its clinical utility as a tumor biomarker and form the framework within which this review should be interpreted. These processes have direct clinical implications for the interpretation of CA19-9 levels, particularly for understanding the dynamic behavior of the marker over time, including false-positive elevations in cholestasis, false-negative results in Lewis-negative individuals, inter-patient variability, and treatment-related fluctuations.

### 2.1. Production

**Figure 1:**
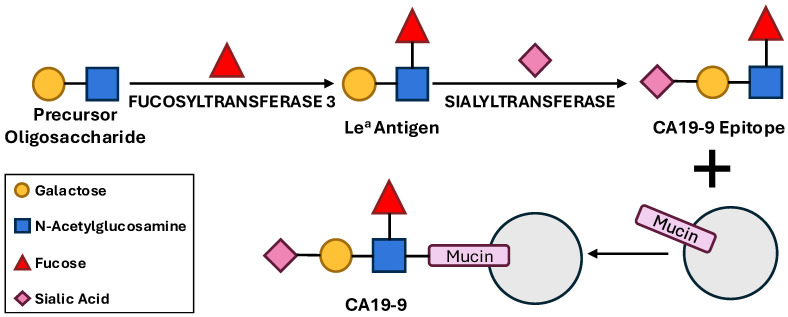
Schematic overview of CA19-9 biosynthesis on mucin-type glycoproteins through sequential glycosylation, involving fucosyltransferases and sialyltransferases. Loss of FUT3 activity results in absent CA19-9 production in Lewis-negative individuals.

CA19-9 is the sialylated form of the Lewis A (Le^a^) antigen, a tetrasaccharide epitope, with the sequence Neu5Acα2,3Galβ1,3(Fucα1,4)GlcNAc, which is carried on high-molecular-weight glycoproteins, primarily mucins [19]. Its production is a multi-step process that begins with the synthesis of mucin glycoproteins, such as MUC1 and MUC16, which serve as protein backbones [16]. The key enzymatic step is the addition of a fucose residue to a precursor oligosaccharide chain by fucosyltransferase 3 (FUT3), which creates the Le^a^ antigen. Subsequently, a sialic acid molecule is added by sialyltransferases (STs), completing the CA19-9 epitope (Figure 1). This pathway explains why individuals with a homozygous recessive Lewis genotype (le/le) lack the necessary FUT3 enzyme and are unable to produce CA19-9 [16]. This genotype is present in approximately 5–10% of the Caucasian population and up to 22% in other populations, leading to false-negative serum CA19-9 levels even in the presence of advanced pancreatic cancer [19]. This genetic requirement is the basis for a major limitation of CA19-9 as a biomarker.

In the bloodstream, the CA19-9 epitope was initially believed to be primarily associated with high-molecular-weight, mucin-like glycoproteins [17]. However, research has identified that CA19-9 is also present on glycolipids embedded in the membranes of secreted vesicles [20]. These carriers include exosomes, small extracellular vesicles involved in intercellular communication [21], and a novel type of larger vesicle, bile globular membranes (BGMs) [20]. The elevated serum levels seen in cancer patients are thought to result from a combination of increased production by tumor cells and an abnormal secretion mechanism that allows these carriers to enter the circulation [20]. This distinction between mucin-bound and vesicle-bound CA19-9 may hold future diagnostic potential, as vesicle-mediated secretion could be more specific to malignant processes.

In healthy individuals, CA19-9 is synthesized in small amounts by the ductal epithelial cells of various organs, including the pancreas, biliary tract, stomach, colon, endometrium, and salivary glands [19]. Its levels in the bloodstream are typically low.

### 2.2. Elevation in Malignant Conditions

PDAC is the malignant condition most strongly and consistently associated with markedly elevated serum CA19-9 levels. In PDAC, the overexpression of the enzymes involved in this glycosylation pathway [22] leads to the abundant production and shedding of CA19-9 into the bloodstream. Single-cell RNA sequencing studies have identified specific cancer cell subtypes as primary producers of CA19-9, contributing to variability in serum levels [22]. This heterogeneity in production may contribute to the variable CA19-9 levels observed among patients with similar tumor burdens. The marker is also found in high concentrations in pancreatic juice [23,24].

CA19-9 is also elevated in bile duct carcinoma (cholangiocarcinoma) and is used in diagnostic and prognostic assessment of intrahepatic cholangiocarcinoma (ICC) [25].

Elevated CA19-9 can be seen in patients with colorectal and gastric cancers, though with lower sensitivity and specificity compared to PDAC. In these contexts, it is more often used as a secondary marker alongside carcinoembryonic antigen (CEA) for monitoring disease rather than for primary diagnosis [26,27].

Less pronounced CA19-9 elevations are reported in a variety of other cancers, including hepatocellular carcinoma, ovarian cancer, endometrial cancer, and lung adenocarcinoma [27,28], with limited clinical utility.

### 2.3. Elevation in Benign Conditions

A major confounding factor in the clinical use of CA19-9 is its elevation in a wide array of non-malignant conditions. These benign elevations are often transient and related to inflammation, obstruction, or tissue injury, which can lead to the leakage of CA19-9 into the bloodstream. The most common benign causes of elevated CA19-9 are diseases of the pancreas [27,29,30,31], biliary tree [27,32], and liver [27,33], the same organs where the antigen is physiologically produced. Other benign conditions that elevate CA19-9 include pulmonary diseases like bronchiectasis and interstitial fibrosis, gynecological disorders such as endometriosis and benign ovarian cysts, and even systemic conditions like diabetes mellitus [27,28]. This underscores the need for caution when interpreting an isolated high CA19-9 value.

### 2.4. Clearance Mechanism

**Figure 2:**
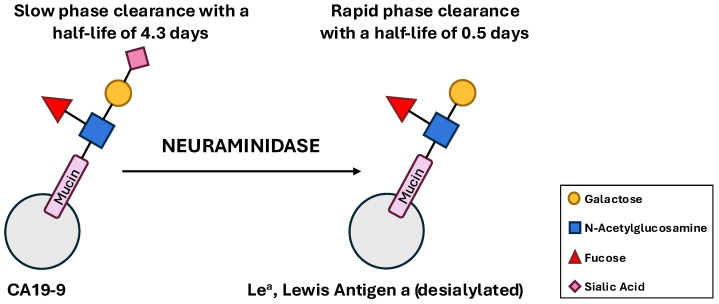
Schematic representation of CA19-9 de-sialylation through enzymatic cleavage of sialic acid by neuraminidases (sialidases). The sialylated form is cleared slowly from the circulation (half-life ≈ 4.3 days), whereas the de-sialylated form is cleared more rapidly (half-life ≈ 0.5 days).

The clearance of circulating CA19-9 is not fully elucidated but is thought to occur primarily in the liver. Early studies revealed that CA19-9 follows a biphasic elimination pattern from the circulation [34,35]. A seminal study by Adachi et al. (1990) [34] using rats injected with CA19-9-bearing molecules from different sources demonstrated a rapid initial phase of clearance followed by a much slower second phase. The half-life of the rapid phase was approximately 1.3 min, while the slow phase had a half-life of 9.8 min for molecules derived from a colon cancer cell line supernatant [34]. Subsequent research in patients following radical resection of intrathoracic malignancies confirmed this biphasic pattern in humans, with a half-life of approximately 0.5 days for the first compartment and 4.3 days for the second [35]. The mechanism underlying this biphasic clearance is believed to be dependent on the degree of sialylation of the CA19-9-bearing molecules [34]. Most circulating glycoproteins are cleared by hepatic lectins after the terminal sialic acid residues are removed. The CA19-9 antigen (sLe^a^) is a sialylated form, while its de-sialylated precursor is the Lewis^a^ (Le^a^) antigen (Figure 2).

Adachi demonstrated that molecules expressing a high level of the Le^a^ epitope were cleared rapidly, corresponding to the first phase. In contrast, molecules with low Le^a^ expression were cleared slowly, corresponding to the second phase. This suggests that the rapid phase represents the removal of partially or fully de-sialylated glycoproteins by the liver, while the slow phase reflects the clearance of intact CA19-9 molecules. The clearance of CA19-9 in the liver is further supported by the clinical observation that benign hepatobiliary diseases, especially those causing cholestasis or biliary obstruction, can lead to markedly elevated CA19-9 levels due to impaired clearance, complicating its interpretation [36].

The serum half-life of CA19-9 is estimated to be relatively short, in the range of days, which is a critical property that underpins its utility as a dynamic marker of tumor activity. This rapid turnover means that changes in tumor burden, whether due to progression or response to therapy, can be reflected in serum CA19-9 levels in a timely manner.

### 2.5. Role in Cancer Pathophysiology

The epidemiological link between pancreatitis and pancreatic cancer is robust and has been recognized for decades [37,38]. Elevated CA19-9 levels in pancreatitis were long considered a consequence of inflammation-induced ductal damage and leakage. However, a landmark study by Engle et al. (2019) fundamentally inverted this cause-and-effect relationship [30].

In a mouse model, the induced expression of CA19-9 alone was sufficient to rapidly trigger severe pancreatitis [30]. This finding provided the first direct evidence that CA19-9 is not just a marker of pancreatic inflammation but a causative agent in its etiology [39]. It remains unclear what pathophysiological events would trigger the increased production of CA19-9 in the first place.

If CA19-9 directly drives disease, then targeting it could be a therapeutic strategy [30,39]. Approaches aimed at inhibiting CA19-9 are being explored, with preclinical studies showing potential for reducing disease severity and progression [40].

By inducing a chronic inflammatory state, CA19-9 fosters a pro-tumorigenic microenvironment. The study by Engle et al. demonstrated that when CA19-9 expression was combined with the presence of the oncogene Kras^G12D^ (a mutation found in over 90% of PDACs), the mice developed highly aggressive and invasive pancreatic cancer [30]. This is consistent with immunohistochemical studies showing that CA19-9 is expressed not only in invasive adenocarcinoma but also in its precursor lesions, pancreatic intraepithelial neoplasia (PanIN), suggesting its involvement from the early stages of the disease [41].

In another study, a subgroup of PDAC patients with normal CA19-9 levels exhibited indolent disease and longer survival [42]. The group had lower circulating glucose levels. In both animals and humans, glucose control measures led to reduced CA19-9 and improved survival, suggesting metabolic interventions as a potential adjuvant therapy for aggressive PDAC [42].

CA19-9 as a ligand for E-selectin, which is expressed on endothelial cells, can mediate the adhesion of circulating tumor cells to the vascular endothelium, a crucial step in the metastatic cascade [16]. The presence of CA19-9 appears to shape the tumor microenvironment. Studies have shown that CA19-9-positive tumors have a higher proportion of mature, M2-like tumor-associated macrophages, which are known to promote an immunosuppressive environment that facilitates tumor growth and immune evasion [22].

## 3. CA19-9 as a Biomarker

A landmark systematic review of pooled data from over 2200 patients established a median sensitivity of 79% and a median specificity of 82% for the diagnosis of pancreatic cancer [43].

These figures show why CA19-9 cannot be used for diagnosis. However, a critical aspect of CA19-9’s performance is its strong correlation with tumor burden. Serum levels tend to increase with advancing tumor stage and size [43,44]. This means that very elevated levels at diagnosis can suggest the presence of metastatic disease in patients otherwise presumed to have resectable disease [17]. For this reason, conventional imaging using CT, Endoscopic Ultrasound (EUS) and/or MRI outperforms CA19-9 in initial diagnosis of pancreatic cancer (Table 1). It has been shown that of the three main imaging modalities, MRI is superior [45].

Due to its insufficient reliability, CA19-9 is also not recommended as a screening tool for pancreatic cancer in the general asymptomatic population [43]. Given these diagnostic limitations, contemporary interest in CA19-9 has shifted from its role as a screening or diagnostic marker to explore its utility in predicting outcomes, monitoring treatment response, and improving prognostic assessment. The following sections therefore focus on the prognostic, predictive, and dynamic roles of CA19-9, and on how its value can be enhanced through integration with other biomarkers and imaging modalities.

### 3.1. Prognostic Role of CA19-9

CA19-9 functions as an independent prognostic biomarker. A strong correlation exists between elevated CA19-9 and more advanced pathological features. Patients with T3 tumors have significantly higher median CA19-9 levels than those with T1/T2 tumors (162 U/mL vs. 41 U/mL). A similar trend is observed for patients with lymph node metastases (N1) compared to node-negative (N0) patients [46]. This relationship extends to the clinical presentation, where patients with locally advanced pancreatic cancer (LAPC) have lower baseline CA19-9 than those with de novo metastatic disease, suggesting distinct biological entities [47].

A high pretreatment or preoperative CA19-9 is an independent predictor of poor overall survival (OS) in patients across all stages of pancreatic cancer. Patients with resectable disease and normal or near normal preoperative CA19-9 levels (<37 U/mL) have a significantly better prognosis compared to those with elevated levels, with median survival times that can be more than doubled [48,49].

In the advanced or metastatic setting, the prognostic value of pretreatment CA19-9 is equally strong. A pooled analysis of six prospective trials of patients receiving gemcitabine-based chemotherapy found that those with baseline CA19-9 levels below the median had a median OS of 8.7 months, compared to just 5.2 months for those with levels above the median [18]. Similarly, a retrospective study separated patients into groups with baseline CA19-9 ≤ 1000 U/mL and >1000 U/mL, finding a stark difference in median OS (14.9 months vs. 7.4 months, respectively), underscoring the marker’s ability to stratify patients into different risk categories from the outset [50]. This prognostic information is independent of other clinical factors like performance status and the presence of metastases [51,52].

### 3.2. Predictive Role of CA19-9

While CA19-9 serves as a prognostic marker independent of treatment, a limited body of data suggests it may also have a potential predictive role that indicates the likelihood of therapeutic response to specific treatments, especially surgical resection. However, it is important to emphasize that most data supporting this application derive from retrospective and observational studies.

The correlation with tumor burden makes CA19-9 a useful tool for assessing the likelihood of resectability. An elevated preoperative CA19-9 level, often cited with a threshold of >100 U/mL in the absence of bile duct obstruction and cholestasis, is frequently associated with occult metastatic disease or local unresectability, even when imaging appears favorable [19,48]. This has led to the incorporation of CA19-9 levels into clinical decision-making, particularly for patients with resectable or borderline-resectable disease. Some guidelines suggest that a “markedly elevated” CA19-9 level may favor the use of neoadjuvant therapy rather than upfront surgery [53]. However, the precise cutoff for what constitutes “markedly elevated” remains a subject of debate, with various studies proposing different thresholds [54].

In advanced pancreatic cancer, a lower baseline CA19-9 (e.g., <1000 U/mL) was correlated with improved survival on first-line gemcitabine-based chemotherapy, suggesting that baseline levels may help stratify risk or inform clinical suspicion [55]. However, randomized trial evidence testing baseline CA19-9 as a true predictive biomarker for differential treatment benefit remains lacking, with available interaction analyses showing no significant modification of treatment effect by baseline CA19-9 levels [53].

### 3.3. Dynamic Role of CA19-9 in Monitoring Pancreatic Cancer

Beyond its baseline utility as a diagnostic, prognostic, or predictive marker, the true strength of CA19-9 lies in its rapid turnover, which allows it to serve as an immediate indicator of changes in tumor burden. Consequently, it provides a robust surrogate for treatment efficacy and residual disease, with applications in both the detection of recurrence and the assessment of therapeutic outcomes.

#### 3.3.1. CA19-9 for Early Detection of Pancreatic Cancer Recurrence

CA19-9 utility in monitoring for recurrence is debated due to limitations, including imprecision and unreliability when using singular static cut-off values of CA19-9 to determine the absence or presence of recurrence, and its absence in Lewis antigen-negative individuals. Clinical practice guidelines often recommend surveillance with a combination of imaging and CA19-9 monitoring [56]. However, the reliance on a static CA19-9 cut-off (typically >37 U/mL) is hampered by suboptimal sensitivity and specificity. False-positive elevations can occur in benign conditions such as cholangitis or pancreatitis, while some true recurrences do not elicit a significant CA19-9 response [57]. Consequently, a confirmed diagnosis of recurrence typically requires radiological evidence.

Current practice mandates confirmation with imaging modalities such as CT or MRI [58]. However, imaging has its own limitations. Post-surgical anatomical changes, scarring, and inflammatory tissue can make it difficult to distinguish between benign postoperative changes and early local recurrence, especially around the surgical bed and major vessels [57]. Furthermore, small-volume peritoneal or distant metastases may be below the resolution limit of standard imaging techniques [59]. This often results in a critical delay between the biochemical signal of recurrence and the initiation of salvage therapy.

A critical consequence of this paradigm is the phenomenon of “biochemical recurrence” preceding “radiological recurrence.” Multiple studies have reported that a rise in CA19-9 levels may occur months before recurrent disease becomes visible on imaging. A study by Azizian et al. found a median lead time of 96 days between a significant CA19-9 elevation and the detection of recurrence by imaging [57]. Similarly, several studies support that CA19-9 elevation can precede clinical or radiological recurrence by 2–6 months [60,61,62]. While in some cases the lead time might have been overestimated due to the standard time interval between imaging, in most cases imaging was performed simultaneously with CA19-9 elevation and failed to detect the recurrence. This lead time represents a potential missed opportunity for earlier therapeutic intervention. However, the anxiety created for patients by the long diagnostic uncertainty is clearly problematic.

To overcome the limitations of a single static cut-off value, recent research has focused on analyzing the dynamics (absolute or percentage changes) of CA19-9 levels over time and how this relates to clinical outcomes for PDAC patients [57,63,64]. Analysis of CA19-9 dynamics has proven more valuable than absolute values at a single timepoint as an indicator of disease status.

While the current evidence highlights the potential of CA19-9 as an early adjunctive indicator of recurrence, isolated CA19-9 elevation in the absence of radiological progression represents a common clinical dilemma, as premature treatment escalation must be weighed against the risk of delayed intervention. Current clinical guidelines do not yet support the use of CA19-9 alone for management decisions, and biochemical changes must always be interpreted in conjunction with imaging and clinical assessment. In this setting, a reasonable management strategy is repeat CA19-9 testing, exclusion of confounding conditions, and alternating or advancing cross-sectional imaging rather than immediate treatment escalation [65].

#### 3.3.2. CA19-9 for Dynamic Evaluation of Therapeutic Response

Following surgical resection, the change in CA19-9 is highly predictive of long-term outcomes. A significant decrease or normalization of postoperative CA19-9 levels is associated with prolonged survival, whereas the failure of CA19-9 to normalize suggests the presence of residual microscopic disease and portends a poor prognosis [48,66]. One study found that the postoperative CA19-9 level was a significant predictor of survival in a multivariate model, independent of preoperative levels and pathologic stage [46]. Another study demonstrated that the failure to normalize preop or postop CA19-9 was associated with a 2.77-fold and 4.03-fold increased risk of death, respectively [67].

Similarly, in patients undergoing chemotherapy for advanced disease, CA19-9 response is a critical prognostic indicator. A pooled analysis of six prospective trials by Bauer et al. (2012) found that a decline in CA19-9 after just two cycles of gemcitabine-based chemotherapy was a significant predictor of improved overall survival [18]. This finding is further supported by other studies that showed patients with an increase of CA19-9 or with a decrease ≤20% following chemotherapy had a poor prognosis [68].

Stemmler et al. (2003) further demonstrated that patients with disease response based on CA19-9 had a significantly longer median survival than those who did not (295 vs. 174 days; *p* = 0.022) [69]. Even among patients whose tumors were progressing according to imaging, those who experienced a CA19-9 reduction survived significantly longer than those who did not (247 vs. 142 days). This highlights that a CA19-9 response, is a more robust predictor of survival than anatomical changes on a CT scan. Subsequent work by Koom et al. (2009) corroborated a strong correlation between CA19-9 decline and radiologic response, with radiologic responders exhibiting greater reductions (93% vs. 72%) than non-responders [70]. More recently, Hahn et al. (2023) showed a significant correlation between the absolute CA19-9 change and both the absolute and relative tumor size reduction on imaging [15].

These findings were further validated in the phase III MPACT trial, which established nab-paclitaxel plus gemcitabine as a standard of care, provided compelling evidence supporting a role for CA19-9 in the metastatic setting [71]. The MPACT trial compared the efficacy and safety of the combination of nab-paclitaxel plus gemcitabine versus gemcitabine monotherapy in patients with metastatic pancreatic cancer [72]. In this analysis, patients who experienced any decrease in CA19-9 at week 8 had a significantly improved median OS compared to those who did not (11.1 vs. 8.0 months; *p* = 0.005). The benefit was particularly striking and statistically significant in the combination therapy arm, where patients with a CA19-9 decline at 8 weeks had a median OS of 13.2 months, versus only 8.3 months for those without a decline/any increase [71]. This study demonstrated that a CA19-9 response could identify patients deriving a substantial survival benefit early in the treatment course. However, it must be noted that these are results in cohorts of patients and that any individual patient may have data confounded by any one of the benign explanations for CA19-9 elevation.

The study also revealed that CA19-9 decline was a more sensitive predictor of survival benefit than early radiographic response. At week 8, 82% of assessable patients in the combination arm had a CA19-9 decrease, and among patients with stable disease by imaging, those who had a concurrent CA19-9 decrease had a markedly better median OS (13.2 months) than those with stable disease and no CA19-9 decrease (8.3 months) [71]. These findings suggest that CA19-9 response can reflect anti-tumor activity early in a clinical trial. Similar findings have been reported in other studies, where a stabilization or decrease in CA19-9 after 6–8 weeks of treatment correlated with significantly better survival than an increase, which was indicative of emerging drug resistance and early treatment failure [50].

#### 3.3.3. CA19-9 for Dynamic Evaluation of Preoperative Chemotherapy

A significant proportion of patients are diagnosed with borderline resectable (BRPC) or locally advanced pancreatic cancer (LAPC), where the tumor has extensive involvement with major blood vessels, making upfront surgery suboptimal or technically unfeasible [73]. This clinical challenge has catalyzed changes in the management of localized pancreatic cancer, moving away from an “upfront surgery” approach towards the increasing use of preoperative, or neoadjuvant, therapy [73].

CA19-9 dynamics during (neo)adjuvant therapy are a significant prognostic factor for patients with pancreatic cancer [74]. A significant drop in CA19-9 can indicate a favorable biological response to treatment, potentially identifying patients who are more likely to achieve a complete resection and have better long-term outcomes. Persistently high or rising CA19-9 may suggest aggressive, resistant disease, prompting a re-evaluation of the surgical strategy [53]. Specifically, the normalization of an initially elevated CA19-9 level (to <37 U/mL) following neoadjuvant therapy has been shown to be a robust predictor of prolonged overall survival [67,75]. Similarly, a large percentage reduction in CA19-9 (e.g., ≥95%) has also been independently associated with favorable survival [76]. This “serological response” offers a critical advantage over imaging, as it can reflect the biological behavior of the tumor and its sensitivity to systemic therapy.

The CA19-9 level measured after the completion of all neoadjuvant therapy, just prior to planned surgery, is arguably one of the most critical data points. An elevated pre-resection CA19-9, especially one that fails to normalize from a high baseline, is a strong negative prognostic marker, portending a high risk of early recurrence and poor survival, even if a complete resection is achieved [67]. This information can be integrated into prognostic scoring systems, such as the PANAMA-score, which combines pre-resection CA19-9 with pathological parameters to improve risk stratification for patients undergoing surgery after preoperative chemotherapy [77].

However, despite the accumulating evidence supporting the utility of CA19-9, its application in the preoperative setting is not yet standardized, and several key questions remain unanswered. The optimal CA19-9 cutoff values for predicting resectability and prognosis are still debated, with different studies proposing various thresholds [78,79]. Furthermore, the precise definition of a “meaningful” serological response is unclear, whether normalization is superior to a specific percentage decline or if the nadir value or rate of decline holds more prognostic weight [67]. Clinical guidelines and practices also vary considerably regarding how to integrate CA19-9 kinetics with imaging and clinical performance status to make the final decision for surgery [73]. Therefore, there is a clear need to refine the clinical application of CA19-9 to optimize patient selection for surgery and improve outcomes in the era of preoperative therapy for pancreatic cancer.

## 4. CA19-9 Enhanced Uses: COMBINATION with Other Markers, Imaging and Genetic Testing

The limitations of CA19-9 as a standalone marker are driving research toward integrated approaches that combine CA19-9 with complementary biomarkers, imaging modalities, and novel genetic testing to provide more comprehensive and accurate disease assessment.

### 4.1. CA19-9 Paired with Other Markers

The most traditional combination is with Carcinoembryonic Antigen (CEA). While CEA itself has poor sensitivity (45%) for pancreatic cancer [80], the simultaneous elevation of both CA19-9 (>75 U/mL) and CEA (>5 ng/mL) dramatically increases the specificity for pancreatic cancer to as high as 99%, albeit at the cost of reduced sensitivity [17]. This combination is also widely tested in other cancers, such as colorectal [81] and gastric malignancies [82].

Macrophage Inhibitory Cytokine-1 (MIC-1) has in some studies shown superior performance to CA19-9 in distinguishing patients with cancer from healthy controls [83,84]. Adding MUC5AC, another mucin, to CA19-9 improved diagnostic accuracy in a large multi-center study [41]. A panel consisting of TIMP1, LRG1, and CA19-9 was shown to better detect early-stage PDAC [85], while IGFBP2 and IGFBP3 have been identified as potential compensatory biomarkers for CA19-9, particularly in early-stage disease [86].

Beyond protein markers, systemic inflammatory markers have gained attention. The combination of CA19-9 with the neutrophil-to-lymphocyte ratio (NLR) and the systemic immune-inflammatory index (SIRI) has been shown to increase diagnostic accuracy for pancreatic cancer compared to any single marker alone [44]. This reflects a growing understanding that the host’s immune response is an integral part of the tumor microenvironment and can be harnessed for diagnostic purposes. A recent diagnostic model for early-stage pancreatic cancer successfully integrated CA19-9 with serum bilirubin levels, demonstrating strong discrimination and clinical utility [87].

The field of liquid biopsy has introduced new classes of biomarkers such as circulating tumor DNA (ctDNA), extracellular vesicles (EVs), and circulating tumor cells (CTCs). These markers offer the potential for high specificity, as they can detect tumor-specific mutations (e.g., KRAS in ctDNA) or carry tumor-derived cargo (e.g., microRNAs in EVs). Studies have shown that monitoring mutant KRAS ctDNA can be comparable to CA19-9 for tracking treatment response in patients who are ctDNA-positive [88].

Similarly, microRNA signatures from small extracellular vesicles (sEVs), when combined with CA19-9, performed better than either modality alone [89]. Exosomal surface proteins, such as CD40 and CD25, are also being explored as complementary markers for both diagnosis and prognosis [7]. However, these advanced tests are currently more complex, costly, and less widely available than the standard CA19-9 immunoassay.

Alterations in metabolism are a hallmark of cancer, and metabolomic profiling has identified biomarker signatures that can distinguish PDAC from chronic pancreatitis with high accuracy, especially when combined with CA19-9 [90].

The ideal future approach will likely involve a multi-analyte panel that integrates the dynamic, quantitative information from CA19-9 with the high-specificity data from genetic, proteomic, and exosomal markers to provide a more holistic and accurate picture of the patient’s disease status [91]. While these combinations are promising, further validation in large-scale studies and clinical trials is essential to fully realize their potential and translate them into routine clinical practice.

### 4.2. CA19-9 Paired with Imaging Modalities

The effective management of patients with PDAC hinges on accurate diagnosis, precise staging, and timely assessment of treatment response. Historically, this has been accomplished through two principal modalities: anatomical imaging, primarily computed tomography (CT), and the measurement of the serum tumor marker CA19-9.

Compared to anatomical imaging such as CT or MRI, CA19-9 offers a more dynamic and functional assessment of tumor activity (Table 2). As discussed, changes in CA19-9 levels can signal treatment response or failure weeks before corresponding changes in tumor size are measurable by RECIST criteria on a CT scan [57,60,61,62,92], While these changes generally need to be validated on anatomical imaging before treatment decisions are made, CA19-9 trends can provide an important early indication of disease progression and guide clinical suspicion.

A pivotal study by Azizian et al. systematically investigated this concept in a cohort of resected PDAC patients [57]. Instead of a fixed threshold, they analyzed the relative change in postoperative CA19-9 values. Using receiver operating characteristic (ROC) curve analysis, they determined that a 2.45-fold elevation from the postoperative baseline value could predict recurrence with 90% sensitivity and 83% specificity in their validation cohort. This approach demonstrated a high positive predictive value (PPV) of 90%, suggesting that a patient with such an increase is highly likely to have recurrent disease, even if imaging is negative. In approximately 60% of patients who relapsed, this significant CA19-9 elevation occurred before imaging could detect the recurrence, with a median lead time of 96 days, equivalent to a typical restaging interval [57].

The clinical significance of this lead time is profound. The same study observed that patients who had a CA19-9 elevation prior to imaging evidence of recurrence had a significantly shorter progression-free survival (PFS) during first-line palliative chemotherapy compared to those whose CA19-9 rose concurrently with imaging findings (median PFS of 97 days vs. 280 days). This suggests that the delay in initiating treatment while waiting for radiological confirmation may allow the disease to progress to a more advanced and less responsive state [57]. Other studies have corroborated the importance of postoperative CA19-9 dynamics. A recent retrospective study highlighted the diagnostic impact of these dynamic changes on predicting recurrence [63], and longitudinal monitoring has also been shown to be predictive of therapeutic responses [64].

To further refine this dynamic approach, Azizian et al. applied a joint model, a sophisticated statistical tool that combines a longitudinal model for the repeated CA19-9 measurements with a survival model for the time-to-recurrence event. This model allows for the estimation of a patient’s individual, real-time probability of remaining recurrence-free, which is updated with each new CA19-9 measurement. In their validation set, this model demonstrated excellent accuracy, with an area under the curve (AUC) of 0.98 for predicting a recurrence event within a two-month interval [57]. Such a model could revolutionize postoperative surveillance by moving away from a one-size-fits-all schedule to a personalized, risk-adapted follow-up. Patients with a low and stable predicted risk of recurrence could have less frequent imaging, while those with a rising risk profile could be monitored more intensively.

Recent advances have moved beyond simple correlations to apply mathematical models to quantify tumor kinetics. Yeh et al. (2023) applied a novel growth and regression model to a large dataset of over 3000 PDAC patients from clinical trials and real-world cohorts [93]. This model estimates a tumor growth rate constant, *g*, using either serial radiographic measurements or CA19-9 values.

This modelling approach provides a sophisticated framework for interpreting serial data, transforming discrete measurements into a continuous variable (*g*) that encapsulates tumor biology and sensitivity to therapy. It reinforces the idea that CA19-9 and imaging are measuring related, but not identical, aspects of tumor progression.

The integrated understanding of how radiological imaging and CA19-9 correlate has several practical implications. First, it allows for earlier assessment of treatment efficacy. A significant CA19-9 decline within the first 4–8 weeks of therapy can provide early reassurance to the patient and clinician, whereas a failure to decline or a rise in CA19-9 can trigger an earlier re-evaluation of the treatment plan. Second, it opens the door to more rational and cost-effective surveillance strategies. The financial burden of cancer care is substantial, and imaging studies are a major contributor. The pilot data from Hahn et al. (2023) [15] provides a rationale for future prospective studies to test an imaging-sparing strategy, where patients with a stable or declining CA19-9 might undergo less frequent CT scans, reducing costs and radiation exposure without compromising care. Third, the prognostic stratification afforded by CA19-9 kinetics and quantitative modelling can help in patient counselling and the design of clinical trials, allowing for the enrichment of study populations or the use of CA19-9 response as an early endpoint [93].

This integrated approach is not without limitations. The inherent biological constraints of CA19-9, particularly its absence in Lewis-negative individuals and its confounding by hyperbilirubinemia, false positives through inflammation and bile duct obstruction/cholestasis must always be considered. There is also a need to standardize the definition of a “CA19-9 response,” with various studies using different thresholds for decline.

### 4.3. CA19-9 Paired with FUT Gene Test

Recent research has investigated the role of fucosyltransferases (FUTs) in enhancing the diagnostic accuracy of CA19-9, revealing a promising approach with significant potential. Although not yet clinically implemented due to the need for extensive validation, this strategy holds the promise of transforming CA19-9 into a more reliable and personalized diagnostic tool. FUTs play a pivotal role in CA19-9 biosynthesis [94]. The final steps are controlled by two key enzymes: the FUT3 enzyme (Lewis enzyme) and the FUT2 enzyme (Secretor enzyme). FUT3 is directly responsible for creating the Lea precursor, while FUT2 competes for the same substrate to produce the H antigen, a precursor for the ABO blood group antigens. The functional status of the genes encoding these two enzymes, FUT3 and FUT2, therefore dictates an individual’s innate ability to synthesize and secrete CA19-9 and provides a rationale for the observed inter-individual variations in CA19-9 levels [95,96,97]:

1. Lewis-negative (le/le): No functional FUT3. No CA19-9 production;
2. Secretors (Se/Se or Se/se) with functional FUT3: FUT2 and FUT3 compete. “Normal” CA19-9 production;
3. Non-secretors (se/se) with functional FUT3: No FUT2 competition. All substrate is available for FUT3, leading to higher baseline CA19-9 levels.

Healthy individuals within specific FUT groups can exhibit varying baseline CA19-9 levels, where higher levels in some groups are not necessarily indicative of disease.

Based on the known biochemistry and analysis of large patient cohorts, four distinct functional groups were defined, each associated with significantly different CA19-9 levels. Consequently, rather than applying a single diagnostic threshold, genotype-specific cut-off values should be considered [98] as summarized in Table 2 below.

**Table 2:** FUT Groups and Upper Cut-Off Values.

Gene Variant	Population Share	Cut-Off Value
FUT3-null	10%	<3 U/mL
FUT-low	34%	34.9 U/mL
FUT-intermediate	34%	41.8 U/mL
FUT-high	22%	89.2 U/mL

Adapted from Dbouk et al., 2023 [98].

When applying the standard uniform cutoff of <36 U/mL, the test achieved a sensitivity of 68.8% but with a relatively low specificity of 91.4%. This means that nearly 9 out of every 100 healthy individuals would have a false-positive result, a rate too high for a screening or early detection setting.

In contrast, when applying the personalized, FUT-stratified cutoffs, the diagnostic specificity soared to 98.9%, meaning only about 1 in 100 healthy individuals would test false-positive. Critically, this massive gain in specificity was achieved with almost no loss in sensitivity, which remained high at 66.7% in the testing set and 65.7% in the validation set of PDAC cases. Note that these studies are performed in healthy populations and do not include “false positive assessment” in patients with biliary or pancreatic ductal obstruction. This remains the major problem in evaluating patients with PDAC.

In high-risk surveillance programs, individuals with a strong family history or genetic predisposition to PDAC undergo regular imaging. A reliable blood test could serve as a valuable adjunct. The high specificity (~99%) of the FUT-stratified could minimize the rate of false-positives that can lead to a cascade of costly and potentially harmful downstream testing [97].

A personalized CA19-9 interpretation has significant prognostic implications. Preliminary evidence from a lung cancer study suggests that when preoperative CA19-9 levels are evaluated using FUT-stratified cutoffs, they have greater power to predict recurrence and mortality after surgery [99]. This FUT-driven approach has the potential to refine risk stratification and inform critical therapeutic decisions.

The FUT gene test represents a promising strategy to potentially refine the clinical interpretation of CA19-9. However, it remains experimental, with limited real-world validation, and no current clinical guidelines recommend routine FUT genotyping. Importantly, while FUT genotyping may explain false-negative CA19-9 results in Lewis-negative individuals, it does not address the most frequent source of false-positive elevations in clinical practice, namely biliary obstruction. Accordingly, the clinical utility of FUT-based stratification requires further validation through prospective and multicenter studies involving larger, more diverse cohorts and real-world clinical applications before it can be adopted for widespread use.

### 4.4. e19-9 as a Substitute for CA19-9

A recent study by Kothari et al. (2025) [100] developed an AI-derived electronic tumor marker, e19-9. This marker, generated from 38 routinely collected serum laboratory values, is designed to predict expected CA19-9 level, serving as a surrogate for patients with non-informative CA19-9. The study evaluated the ability of these 38 laboratory values, determined at diagnosis and after treatment, to predict key clinical outcomes.

A decline in e19-9 of at least 50% and a post-treatment e19-9 < 100 were both predictors of completing therapy and surgery. In multivariable analysis, a post-treatment e19-9 < 100 was independently associated with 19.3-fold increased odds of completing treatment and surgery. Furthermore, e19-9 dynamics also predicted development of metastatic disease prior to surgery. Patients who progressed to metastatic disease experienced a median increase in e19-9 of 26.4%, while those who did not had a median decrease of 52.7% [100].

The e19-9 marker demonstrated a profound correlation with survival. Patients with a post-treatment e19-9 < 100 had dramatically improved median OS (60 vs. 16 months). This prognostic power held even among the subset of patients who successfully underwent surgery. In multivariable Cox proportional hazards models, a post-treatment e19-9 < 100 is an independent predictor of improved survival (HR 0.49, *p* = 0.04) [100].

From a biological standpoint e19-9 does not reflect direct tumor-specific antigen production, but rather likely captures a systemic host-response, integrating features of inflammation (e.g., neutrophil-to-lymphocyte ratio), nutritional status (e.g., albumin), and organ function (e.g., bilirubin, renal function), which collectively paint a more comprehensive picture of a patient’s physiological response to their cancer and its treatment than any single marker could Accordingly, its correlation with tumor burden is indirect and mediated through the physiological effects of malignancy rather than through tumor-derived glycan expression [100].

The current evidence supporting e19-9 is derived from a single retrospective study, and independent external validation is lacking. Although e19-9 requires no specialized testing beyond routinely collected laboratory data and could be readily integrated into electronic health record systems, its clinical use remains experimental. Prospective, multicenter studies are required to confirm its real-world performance, assess any potential bias or performance drift over time.

Nevertheless, the potential clinical impact is substantial. For patients with non-informative CA19-9, e19-9 could enable longitudinally monitoring of treatment response, enabling the kind of adaptive therapy strategies that are currently reserved for CA19-9 producers. The primary advantage of e19-9 over other emerging biomarkers is its unparalleled accessibility and cost-effectiveness.

## 5. Discussion

The clinical journey of a patient with pancreatic cancer is marked by critical decision points, from diagnosis and staging to treatment selection and surveillance. Throughout this journey, both imaging and biomarkers play a pivotal role in guiding management. This review has synthesized the available body of evidence surrounding CA19-9, highlighting its dual identity: a sub-optimal biomarker for initial diagnosis but an exceptionally valuable one for the longitudinal monitoring of established disease.

Imaging provides the essential anatomical roadmap for staging and assessing resectability, while CA19-9 kinetics offer a dynamic, real-time window into the tumor’s biological response to therapy. The synergy between the two modalities is evident when their findings align. A partial response on imaging accompanied by a precipitous drop in CA19-9 is a clear signal of treatment efficacy. However, it is in the instances of discordance where the integrated approach is most valuable. As demonstrated by Stemmler et al. (2003) [69], a falling CA19-9 in the face of stable or even slowly progressing disease on CT is not a contradictory finding but rather a positive prognostic indicator. It suggests that while the therapy may not be cytotoxic enough to shrink the bulky, fibrotic tumor mass, it is effectively controlling the biologically active component of the cancer, leading to improved survival. Conversely, a rising CA19-9 despite stable imaging is an ominous sign, often heralding imminent clinical and radiologic progression, and should prompt earlier imaging, although a change in therapy based on CA19-9 alone cannot be recommended due to the high rates of CA19-9 elevation in patients with biliary and pancreatic duct anomalies.

This finding is supported by several key studies that concur that “biochemical recurrence” as detected through changes in CA19 levels precedes “radiological recurrence” confirmed by imaging with a lead time ranging from 2–6 months [18,57,60,61,63,64,70]. This is significant because it means that although current clinical guidelines do not yet support this approach, in the future CA19-9 could potentially be used on its own in patients with stable or declining CA19-9 levels post-treatment or post-operatively [15]. With the development of the AI-derived e19-9 substitute for CA19-9, individuals incapable of naturally producing CA19-9 can still be given the same level of care as those being monitored using CA19-9 [100].

The fundamental properties of CA19-9—its correlation with tumor burden and its rapid production and clearance dynamics—are what make it so effective in the post-diagnostic setting. Unlike anatomical imaging, which provides static snapshots in time, serial CA19-9 measurements offer a continuous, real-time assessment of the tumor’s biological activity. This allows clinicians to gauge prognostic risk, assess the efficacy of systemic therapy with greater sensitivity than imaging alone, and detect disease recurrence at its earliest biochemical stages. The clinical implication is significant: by tracking the trend of CA19-9, clinicians can make more informed and timely decisions, such as escalating care for patients with a poor prognostic profile, continuing effective therapy, or switching to a different regimen at the first sign of treatment failure, potentially before the patient suffers irreversible clinical decline.

However, it is crucial to acknowledge the limitations of the existing evidence and the nuances of clinical application. Many studies evaluating CA19-9 are retrospective, and there is a lack of standardization in the cut-off values used to define a “significant” change or a “high” level, which can lead to variability in interpretation [101]. In addition, intra-individual biological variability, and the lack of standardized timing for blood sampling may introduce further variability in CA19-9 measurements and should be considered when interpreting longitudinal trends [102]. In routine clinical practice, the interpretation of dynamic CA19-9 changes must always account for major confounders such as cholestasis, biliary obstruction, and inflammatory conditions including pancreatitis, all of which can cause substantial fluctuations independent of tumor burden and significantly limit day-to-day interpretability. To mitigate these effects CA19-9 levels can be re-evaluated after biliary decompression, which often leads to a parallel decrease in CA19-9 and bilirubin levels, unrelated to tumor burden [103]. Establishing a post-drainage baseline provides a more accurate reference for subsequent tumor-specific monitoring. To further correct for cholestasis-related distortion without invasive procedures, biochemical normalization strategies have been proposed, including ratios between CA19-9 and liver enzymes, which may help disentangle tumor-related production from obstruction-related elevation [104]. In conclusion, the most prudent clinical practice involves interpreting CA19-9 levels not in isolation, but as part of a comprehensive assessment that includes the patient’s clinical status, imaging results, and other laboratory data. The emphasis should always be on the trend over time rather than on a single absolute value.

This review of available evidence highlights that the limitations of CA19-9 as a diagnostic marker have adversely impacted its clinical use, leading to its exclusion as a registrational endpoint in clinical trials. While the limitations around its use in diagnosis of PDAC are valid, new technologies are being developed to overcome these limitations. Of particular interest is the FUT gene test which not only differentiates CA19-9 producers from non-producers but applies genetically based personalized thresholds that improve CA19-9 diagnostic performance and enhance its established value as a monitoring biomarker [98]. Although the FUT genotype test is not currently available in clinical practice, its potential to improve diagnostic accuracy by accounting for genetic variability in CA19-9 levels is driving further research to move toward clinical implementation. Even as research is ongoing on improving the diagnostic performance of CA19-9, there is sufficient and very strong evidence to support the clinical significance and use of CA19-9 across the care continuum for PDAC.

The future of biomarker-guided management in PDAC is moving towards personalization and integration. The limitations of CA19-9, particularly in the Lewis-negative population and for early detection, are being actively addressed including through the development of multi-analyte panels. The integration of CA19-9 with highly specific markers from liquid biopsies, such as ctDNA and EVs, holds the promise of a new paradigm in patient care. Such panels could one day provide not only a measure of tumor burden (from CA19-9) but also real-time information on the tumor’s genetic landscape, resistance mechanisms, and metastatic potential. Prospective validation of these emerging biomarker panels in large, well-designed clinical trials is the critical next step to bring them into routine clinical practice.

## 6. Conclusions

In the challenging landscape of pancreatic ductal adenocarcinoma management, CA19-9 remains an imperfect but important tool. Its well-documented limitations for screening and diagnosis are counterbalanced by its proven utility as a dynamic biomarker for prognosis and therapeutic monitoring. The ability of CA19-9 to reflect real-time changes in tumor activity provides clinicians with a cost-effective and widely accessible means to guide treatment decisions, predict outcomes, and conduct surveillance for recurrence. These insights highlight its potential to serve as a timely indicator of disease progression and therapeutic efficacy in clinical trial research.

Beyond its clinical utility, emerging evidence highlights a broader biological significance of CA19-9, reframing it from a passive biomarker to a potential therapeutic target. While its role as a driver of inflammation and cancer progression is intriguing, the primary focus remains its role in improving patient care. CA19-9’s dual capacity to inform therapeutic strategies and serve as a marker of disease activity ensures its continued relevance in both clinical settings and research, paving the way for future innovations in pancreatic cancer management.

## Figures and Tables

**Table 1:** Performance of Conventional Imaging in Diagnosing Pancreatic Adenocarcinoma.

Imaging Modality	Sensitivity	Specificity	Diagnostic Accuracy
MRI	93% (95% CI = 88–96)	89% (95% CI = 82–94)	90% (95% CI = 86–94)
CT	90% (95% CI = 87–93)	87% (95% CI = 79–93)	89% (95% CI = 85–93)
EUS	91% (95% CI = 87–94)	86% (95% CI = 81–91)	89% (95% CI = 87–92)

Adapted from Toft et al., 2017 [45].

## Data Availability

This is a review article and does not involve the creation of new data. All data supporting the findings of this study are derived from previously published sources, which are appropriately cited within the article.

## Abbreviations

The following abbreviations are used in this manuscript:

AUC	Area Under the Curve
BRPC	Borderline Resectable Pancreatic Cancer
CA19-9	Carbohydrate Antigen 19-9
CEA	Carcinoembryonic Antigen
CT	Computer Tomography
CTC	Circulating Tumor Cells
ctDNA	Circulating Tumor DNA
e19-9	Electronic 19-9
ESMO	European Society of Medical Oncology
EUS	Endoscopic Ultrasound
EV	Extracellular Vesicle
FUT	Fucosyltransferases
LAPC	Locally Advanced Pancreatic Cancer
Lea	Lewisa Antigen
MRI	Magnetic Resonance Imaging
NLR	Neutrophil-to-Lymphocyte Ratio
OS	Overall Survival
PDAC	Pancreatic Ductal Adenocarcinoma
PFS	Progression-Free Survival
PPV	Positive Predictive Value
ROC	Receiver Operator Characteristic
SIRI	Systemic Immune-inflammatory Index

## References

[1] Ferlay J., Ervik M., Lam F., Laversanne M., Colombet M., Mery L., Pineros M., Znaor A., Soerjomataram I., Bray F. (2024). Global Cancer Observatory: Cancer Today (Version 1.1).

[2] Kleeff J., Korc M., Apte M., La Vecchia C., Johnson C.D., Biankin A.V., Neale R.E., Tempero M., Tuveson D.A., Hruban R.H. (2016). Pancreatic Cancer. Nat. Rev. Dis. Primers.

[3] Orth M., Metzger P., Gerum S., Mayerle J., Schneider G., Belka C., Schnurr M., Lauber K. (2019). Pancreatic Ductal Adenocarcinoma: Biological Hallmarks, Current Status, and Future Perspectives of Combined Modality Treatment Approaches. Radiat. Oncol..

[4] Principe D.R., Underwood P.W., Korc M., Trevino J.G., Munshi H.G., Rana A. (2021). The Current Treatment Paradigm for Pancreatic Ductal Adenocarcinoma and Barriers to Therapeutic Efficacy. Front. Oncol..

[5] Song H., Hsu J., Lin X., Ogawa S., Pantazopoulou V., Peck K., Bottomley C., Okhovat S., Stamp M., Curtis K. (2024). Abstract 3937: Investigating the Role of Fibulin 3 in Pancreatic Tumorigenesis. Cancer Res..

[6] Hidalgo M., Cascinu S., Kleeff J., Labianca R., Löhr J.-M., Neoptolemos J., Real F.X., Van Laethem J.-L., Heinemann V. (2015). Addressing the Challenges of Pancreatic Cancer: Future Directions for Improving Outcomes. Pancreatology.

[7] David P., Kouhestani D., Hansen F.J., Paul S., Czubayko F., Karabiber A., Weisel N., Klösch B., Merkel S., Ole-Baur J. (2025). Exosomal CD40, CD25, and Serum CA19-9 as Combinatory Novel Liquid Biopsy Biomarker for the Diagnosis and Prognosis of Patients with Pancreatic Ductal Adenocarcinoma. Int. J. Mol. Sci..

[8] Wen Y.-R., Lin X.-W., Zhou Y.-W., Xu L., Zhang J.-L., Chen C.-Y., He J. (2024). N-Glycan Biosignatures as a Potential Diagnostic Biomarker for Early-Stage Pancreatic Cancer. World J. Gastrointest. Oncol..

[9] Kunovsky L., Tesarikova P., Kala Z., Kroupa R., Kysela P., Dolina J., Trna J. (2018). The Use of Biomarkers in Early Diagnostics of Pancreatic Cancer. Can. J. Gastroenterol. Hepatol..

[10] Ren S., Song L.-N., Zhao R., Tian Y., Wang Z.-Q. (2025). Serum Exosomal Hsa-Let-7f-5p: A Potential Diagnostic Biomarker for Metastatic Pancreatic Cancer Detection. World J. Gastroenterol..

[11] Khomiak A., Brunner M., Kordes M., Lindblad S., Miksch R.C., Öhlund D., Regel I. (2020). Recent Discoveries of Diagnostic, Prognostic and Predictive Biomarkers for Pancreatic Cancer. Cancers.

[12] Winter J.M., Yeo C.J., Brody J.R. (2013). Diagnostic, Prognostic, and Predictive Biomarkers in Pancreatic Cancer. J. Surg. Oncol..

[13] Conroy T., Pfeiffer P., Vilgrain V., Lamarca A., Seufferlein T., O’Reilly E.M., Hackert T., Golan T., Prager G., Haustermans K. (2023). Pancreatic Cancer: ESMO Clinical Practice Guideline for Diagnosis, Treatment and Follow-Up. Ann. Oncol..

[14] Bilreiro C., Andrade L., Santiago I., Marques R.M., Matos C. (2024). Imaging of Pancreatic Ductal Adenocarcinoma—An Update for All Stages of Patient Management. Eur. J. Radiol. Open.

[15] Hahn A., Travis B., Hunter D., Colorafi A., Regan C., Bay C., Koo P., Dragovich T., Choti M.A., Kundranda M.N. (2023). Association of CA19-9 and Tumor Size in Treatment Monitoring for Patients with Metastatic Pancreatic Ductal Adenocarcinoma (PDAC). J. Clin. Oncol..

[16] Luo G., Jin K., Deng S., Cheng H., Fan Z., Gong Y., Qian Y., Huang Q., Ni Q., Liu C. (2021). Roles of CA19-9 in Pancreatic Cancer: Biomarker, Predictor and Promoter. Biochim. Biophys. Acta (BBA) Rev. Cancer.

[17] Steinberg W.M., Gelfand R., Anderson K.K., Glenn J., Kurtzman S.H., Sindelar W.F., Toskes P.P. (1986). Comparison of the Sensitivity and Specificity of the CA19-9 and Carcinoembryonic Antigen Assays in Detecting Cancer of the Pancreas. Gastroenterology.

[18] Bauer T.M., El-Rayes B.F., Li X., Hammad N., Philip P.A., Shields A.F., Zalupski M.M., Bekaii-Saab T. (2013). Carbohydrate Antigen 19-9 Is a Prognostic and Predictive Biomarker in Patients with Advanced Pancreatic Cancer Who Receive Gemcitabine-Containing Chemotherapy. Cancer.

[19] Scarà S., Bottoni P., Scatena R., Scatena R. (2015). CA 19-9: Biochemical and Clinical Aspects. Advances in Cancer Biomarkers: From Biochemistry to Clinic for a Critical Revision.

[20] Uozumi N., Gao C., Yoshioka T., Nakano M., Moriwaki K., Nakagawa T., Masuda T., Tanabe M., Miyoshi E. (2010). Identification of a Novel Type of CA19-9 Carrier in Human Bile and Sera of Cancer Patients: An Implication of the Involvement in Nonsecretory Exocytosis. J. Proteome Res..

[21] Ogata-Kawata H., Izumiya M., Kurioka D., Honma Y., Yamada Y., Furuta K., Gunji T., Ohta H., Okamoto H., Sonoda H. (2014). Circulating Exosomal microRNAs as Biomarkers of Colon Cancer. PLoS ONE.

[22] Zhang D., Cui F., Zheng K., Li W., Liu Y., Wu C., Peng L., Yang Z., Chen Q., Xia C. (2024). Single-Cell RNA Sequencing Reveals the Process of CA19-9 Production and Dynamics of the Immune Microenvironment between CA19-9 (+) and CA19-9 (−) PDAC. Chin. Med. J..

[23] Grønborg M., Bunkenborg J., Kristiansen T.Z., Jensen O.N., Yeo C.J., Hruban R.H., Maitra A., Goggins M.G., Pandey A. (2004). Comprehensive Proteomic Analysis of Human Pancreatic Juice. J. Proteome Res..

[24] Wang J., Raimondo M., Guha S., Chen J., Diao L., Dong X., Wallace M.B., Killary A.M., Frazier M.L., Woodward T.A. (2014). Circulating microRNAs in Pancreatic Juice as Candidate Biomarkers of Pancreatic Cancer. J. Cancer.

[25] Jia Y., Wan M., Shen Y., Wang J., Luo X., He M., Bai R., Xiao W., Zhang X., Ruan J. (2025). Predictive Nomogram Integrating Radiomics and Multi-Omics for Improved Prognosis-Model in Cholangiocarcinoma. Clin. Transl. Med..

[26] Burdick M.D., Harris A., Reid C.J., Iwamura T., Hollingsworth M.A. (1997). Oligosaccharides Expressed on MUC1 Produced by Pancreatic and Colon Tumor Cell Lines*. J. Biol. Chem..

[27] Ito S., Gejyo F. (1999). Elevation of Serum CA19-9 Levels in Benign Diseases. Intern. Med..

[28] Zhuang Y., Cai Q., Hu X., Huang H. (2024). Elevated Serum CA199 Levels in Patients Suffering Type 2 Diabetes vs. Various Types of Cancer. BMC Endocr. Disord..

[29] Uno K., Azuma T., Nakajima M., Yasuda K., Hayakumo T., Mukai H., Sakai T., Kawai K. (2000). Clinical Significance of Cathepsin E in Pancreatic Juice in the Diagnosis of Pancreatic Ductal Adenocarcinoma. J. Gastroenterol. Hepatol..

[30] Engle D.D., Tiriac H., Rivera K.D., Pommier A., Whalen S., Oni T.E., Alagesan B., Lee E.J., Yao M.A., Lucito M.S. (2019). The Glycan CA19-9 Promotes Pancreatitis and Pancreatic Cancer in Mice. Science.

[31] Zhao Y.-S., Su P., Li Z. (2024). Gastrointestinal: A Cystic-Solid Pancreatic Mass: Pancreatitis or Adenocarcinoma?. J. Gastroenterol. Hepatol..

[32] Chahine A., Tavangar A., Lin A., Jariwalla N., Ho J., Ji S., Samarasena J. (2023). S2234 Unmasking Obstructive Jaundice: A Rare Presentation of MALT Lymphoma. Off. J. Am. Coll. Gastroenterol. ACG.

[33] Drenth J.P.H., Chrispijn M., Nagorney D.M., Kamath P.S., Torres V.E. (2010). Medical and Surgical Treatment Options for Polycystic Liver Disease. Hepatology.

[34] Adachi M., Sekine T., Umemoto A., Tsukikawa S., Imai K., Yachi A. (2009). Mechanism of Clearance of Circulating CA19–9 in Rats. Tumor Biol..

[35] Yoshimasu T., Maebeya S., Suzuma T., Bessho T., Tanino H., Arimoto J., Sakurai T., Naito Y. (1999). Disappearance Curves for Tumor Markers after Resection of Intrathoracic Malignancies. Int. J. Biol. Markers.

[36] Pleskow D.K., Berger H.J., Gyves J., Allen E., McLean A., Podolsky D.K. (1989). Evaluation of a Serologic Marker, CA19-9, in the Diagnosis of Pancreatic Cancer. Ann. Intern. Med..

[37] Guerra C., Collado M., Navas C., Schuhmacher A.J., Hernández-Porras I., Cañamero M., Rodriguez-Justo M., Serrano M., Barbacid M. (2011). Pancreatitis-Induced Inflammation Contributes to Pancreatic Cancer by Inhibiting Oncogene-Induced Senescence. Cancer Cell.

[38] Lowenfels A.B., Maisonneuve P., Cavallini G., Ammann R.W., Lankisch P.G., Andersen J.R., DiMagno E.P., Andren-Sandberg A., Domellof L. (1993). Pancreatitis and the Risk of Pancreatic Cancer. N. Engl. J. Med..

[39] Negoi I., Beuran M., Hostiuc S., Sartelli M., El-Hussuna A., de-Madaria E. (2019). Glycosylation Alterations in Acute Pancreatitis and Pancreatic Cancer: CA19-9 Expression Is Involved in Pathogenesis and Maybe Targeted by Therapy. Ann. Transl. Med..

[40] Langbein T., Weber W.A., Eiber M. (2019). Future of Theranostics: An Outlook on Precision Oncology in Nuclear Medicine. J. Nucl. Med..

[41] Kaur S., Smith L.M., Patel A., Menning M., Watley D.C., Malik S.S., Krishn S.R., Mallya K., Aithal A., Sasson A.R. (2017). A Combination of MUC5AC and CA19-9 Improves the Diagnosis of Pancreatic Cancer: A Multicenter Study. Off. J. Am. Coll. Gastroenterol. ACG.

[42] Zhu X., Xiao Z., Liu H., Zhang P., Deng S., Ding L., Feng J., Luo J., Ni Q., Luo G. (2024). Pancreatic Cancer: An Exocrine Tumor With Endocrine Characteristics. Ann. Surg..

[43] Goonetilleke K.S., Siriwardena A.K. (2007). Systematic Review of Carbohydrate Antigen (CA 19-9) as a Biochemical Marker in the Diagnosis of Pancreatic Cancer. Eur. J. Surg. Oncol. (EJSO).

[44] Li M., Dong Z., Zhang X., Xue S., Wang B. (2021). Analysis of Levels and Clinical Value of CA19-9, NLR and SIRI in Patients with Pancreatic Cancer with Different Clinical Features. Cell. Mol. Biol..

[45] Toft J., Hadden W.J., Laurence J.M., Lam V., Yuen L., Janssen A., Pleass H. (2017). Imaging Modalities in the Diagnosis of Pancreatic Adenocarcinoma: A Systematic Review and Meta-Analysis of Sensitivity, Specificity and Diagnostic Accuracy. Eur. J. Radiol..

[46] Ferrone C.R., Finkelstein D.M., Thayer S.P., Muzikansky A., Castillo C.F., Warshaw A.L. (2006). Perioperative CA19-9 Levels Can Predict Stage and Survival in Patients With Resectable Pancreatic Adenocarcinoma. J. Clin. Oncol..

[47] Gonzalez Conchas G.A., O’Kane G.M., Denroche R.E., Jang G.H., Fischer S., Dodd A., Picardo S.L., Holter S., Wilson J., Zogopoulos G. (2024). Characterizing the Genomic Landscape of Locally Advanced Pancreatic Cancer. J. Clin. Oncol..

[48] Ballehaninna U.K., Chamberlain R.S. (2011). The Clinical Utility of Serum CA 19-9 in the Diagnosis, Prognosis and Management of Pancreatic Adenocarcinoma: An Evidence Based Appraisal. J. Gastrointest. Oncol..

[49] Katz M.H.G., Varadhachary G.R., Fleming J.B., Wolff R.A., Lee J.E., Pisters P.W.T., Vauthey J.-N., Abdalla E.K., Sun C.C., Wang H. (2010). Serum CA 19-9 as a Marker of Resectability and Survival in Patients with Potentially Resectable Pancreatic Cancer Treated with Neoadjuvant Chemoradiation. Ann. Surg. Oncol..

[50] Pelzer U., Hilbig A., Stieler J., Sinn M., Bahra M., Dörken B., Riess H. (2013). Value of Carbohydrate Antigen 19-9 in Predicting Response and Therapy Control in Patients with Metastatic Pancreatic Cancer Undergoing First-Line Therapy. Front. Oncol..

[51] Cetin S., Dede I. (2020). Prognostic Value of the Neutrophil-to-Lymphocyte Ratio and Carbohydrate Antigen 19-9 in Estimating Survival in Patients with Metastatic Pancreatic Cancer. J. Cancer Res. Ther..

[52] Ermiah E., Eddfair M., Abdulrahman O., Elfagieh M., Jebriel A., Al-Sharif M., Assidi M., Buhmeida A. (2022). Prognostic Value of Serum CEA and CA199 Levels in Pancreatic Ductal Adenocarcinoma. Mol. Clin. Oncol..

[53] Doppenberg D., van Dam J.L., Han Y., Bonsing B.A., Busch O.R., Festen S., van der Harst E., de Hingh I.H., Homs M.Y.V., Kwon W. (2023). Predictive Value of Baseline Serum Carbohydrate Antigen 19-9 Level on Treatment Effect of Neoadjuvant Chemoradiotherapy in Patients with Resectable and Borderline Resectable Pancreatic Cancer in Two Randomized Trials. Br. J. Surg..

[54] Hartwig W., Strobel O., Hinz U., Fritz S., Hackert T., Roth C., Büchler M.W., Werner J. (2013). CA19-9 in Potentially Resectable Pancreatic Cancer: Perspective to Adjust Surgical and Perioperative Therapy. Ann. Surg. Oncol..

[55] Hammad N., Heilbrun L.K., Philip P.A., Shields A.F., Zalupski M.M., Venkatramanamoorthy R., El-Rayes B.F. (2010). CA19-9 as a Predictor of Tumor Response and Survival in Patients with Advanced Pancreatic Cancer Treated with Gemcitabine Based Chemotherapy. Asia-Pac. J. Clin. Oncol..

[56] Seufferlein T., Bachet J.B., Van Cutsem E., Rougier P. (2012). Pancreatic Adenocarcinoma: ESMO–ESDO Clinical Practice Guidelines for Diagnosis, Treatment and Follow-Up†. Ann. Oncol..

[57] Azizian A., Rühlmann F., Krause T., Bernhardt M., Jo P., König A., Kleiß M., Leha A., Ghadimi M., Gaedcke J. (2020). CA19-9 for Detecting Recurrence of Pancreatic Cancer. Sci. Rep..

[58] Chu L.C., Fishman E.K. (2024). Pancreatic Ductal Adenocarcinoma Staging: A Narrative Review of Radiologic Techniques and Advances. Int. J. Surg..

[59] Rose D.M., Delbeke D., Beauchamp R.D., Chapman W.C., Sandler M.P., Sharp K.W., Richards W.O., Wright J.K., Frexes M.E., Pinson C.W. (1999). 18Fluorodeoxyglucose-Positron Emission Tomography in the Management of Patients With Suspected Pancreatic Cancer. Ann. Surg..

[60] Meira-Júnior J.D.D., Costa T.N., Montagnini A.L., Nahas S.C., Jukemura J. (2022). Elevated CA 19-9 in an asymptomatic patient: What does it mean?. Arq. Bras. Cir. Dig..

[61] Li J., Li Z., Kan H., Sun Z., Xing J., Cheng Y., Bai C. (2019). CA19-9 Elevation as an Indication to Start Salvage Treatment in Surveillance after Pancreatic Cancer Resection. Pancreatology.

[62] Lee T., Teng T., Shelat V. (2020). Carbohydrate Antigen 19-9—Tumor Marker: Past, Present, and Future. World J. Gastrointest. Surg..

[63] Imamura H., Tomimaru Y., Kobayashi S., Yamada D., Noda T., Takahashi H., Doki Y., Eguchi H. (2024). Diagnostic Impact of Postoperative CA19-9 Dynamics on Pancreatic Cancer Recurrence: A Single-Institution Retrospective Study. Updates Surg..

[64] Watanabe F., Suzuki K., Tamaki S., Abe I., Endo Y., Takayama Y., Ishikawa H., Kakizawa N., Saito M., Futsuhara K. (2020). Longitudinal Monitoring of KRAS-Mutated Circulating Tumor DNA Enables the Prediction of Prognosis and Therapeutic Responses in Patients with Pancreatic Cancer. PLoS ONE.

[65] Nong M.Z., Dove D., Fischer D.A., Hourdequin K.C., Ripple G.H., Amin M.A., McGrath E.B., Zaki B.I., Smith K.D., Brooks G.A. (2024). Surveillance with Serial Imaging and CA 19-9 Tumor Marker Testing After Resection of Pancreatic Cancer: A Single-Center Retrospective Study. Am. J. Clin. Oncol..

[66] Kiczmer P., Seńkowska A.P., Szydło B., Świętochowska E., Ostrowska Z. (2017). Assessing the Merits of Existing Pancreatic Cancer Biomarkers. Nowotwory. J. Oncol..

[67] Tsai S., George B., Wittmann D., Ritch P.S., Krepline A.N., Aldakkak M., Barnes C.A., Christians K.K., Dua K., Griffin M. (2020). Importance of Normalization of CA19-9 Levels Following Neoadjuvant Therapy in Patients with Localized Pancreatic Cancer. Ann. Surg..

[68] Ziske C., Schlie C., Gorschluter M., Glasmacher A., Mey U., Strehl J., Sauerbruch T., Schmidt-Wolf I.G.H. (2003). Prognostic Value of CA 19-9 Levels in Patients with Inoperable Adenocarcinoma of the Pancreas Treated with Gemcitabine. Br. J. Cancer.

[69] Stemmler J., Stieber P., Szymala A.M., Schalhorn A., Schermuly M.M., Wilkowski R., Helmberger T., Lamerz R., Stoffregen C., Niebler K. (2003). Are Serial CA 19-9 Kinetics Helpful in Predicting Survival in Patients with Advanced or Metastatic Pancreatic Cancer Treated with Gemcitabine and Cisplatin?. Onkologie.

[70] Koom W.S., Seong J., Kim Y.B., Pyun H.O., Song S.Y. (2009). CA 19-9 as a Predictor for Response and Survival in Advanced Pancreatic Cancer Patients Treated With Chemoradiotherapy. Int. J. Radiat. Oncol. Biol. Phys..

[71] Chiorean E.G., Von Hoff D.D., Reni M., Arena F.P., Infante J.R., Bathini V.G., Wood T.E., Mainwaring P.N., Muldoon R.T., Clingan P.R. (2016). CA19-9 Decrease at 8 Weeks as a Predictor of Overall Survival in a Randomized Phase III Trial (MPACT) of Weekly Nab-Paclitaxel plus Gemcitabine versus Gemcitabine Alone in Patients with Metastatic Pancreatic Cancer. Ann. Oncol..

[72] Von Hoff D.D., Ervin T., Arena F.P., Chiorean E.G., Infante J., Moore M., Seay T., Tjulandin S.A., Ma W.W., Saleh M.N. (2013). Increased Survival in Pancreatic Cancer with Nab-Paclitaxel plus Gemcitabine. N. Engl. J. Med..

[73] Stoop T.F., Theijse R.T., Seelen L.W.F., Groot Koerkamp B., van Eijck C.H.J., Wolfgang C.L., van Tienhoven G., van Santvoort H.C., Molenaar I.Q., Wilmink J.W. (2024). Preoperative Chemotherapy, Radiotherapy and Surgical Decision-Making in Patients with Borderline Resectable and Locally Advanced Pancreatic Cancer. Nat. Rev. Gastroenterol. Hepatol..

[74] Ye C., Sadula A., Ren S., Guo X., Yuan M., Yuan C., Xiu D. (2020). The Prognostic Value of CA19-9 Response after Neoadjuvant Therapy in Patients with Pancreatic Cancer: A Systematic Review and Pooled Analysis. Cancer Chemother. Pharmacol..

[75] Igarashi T., Yamada S., Hoshino Y., Murotani K., Baba H., Takami H., Yoshioka I., Shibuya K., Kodera Y., Fujii T. (2023). Prognostic Factors in Conversion Surgery Following Nab-Paclitaxel with Gemcitabine and Subsequent Chemoradiotherapy for Unresectable Locally Advanced Pancreatic Cancer: Results of a Dual-Center Study. Ann. Gastroenterol. Surg..

[76] Uemura K., Kondo N., Sudo T., Sumiyoshi T., Shintakuya R., Okada K., Baba K., Harada T., Murakami Y., Takahashi S. (2024). Long-Term Outcomes of Neoadjuvant Gemcitabine, Nab-Paclitaxel, and S1 (GAS) in Borderline Resectable Pancreatic Cancer with Arterial Contact: Results from a Phase II Trial. J. Hepato-Biliary-Pancreat. Sci..

[77] Hank T., Sandini M., Ferrone C.R., Ryan D.P., Mino-Kenudson M., Qadan M., Wo J.Y., Klaiber U., Weekes C.D., Weniger M. (2022). A Combination of Biochemical and Pathological Parameters Improves Prediction of Postresection Survival After Preoperative Chemotherapy in Pancreatic Cancer: The PANAMA-Score. Ann. Surg..

[78] Hasebe R., Watanabe F., Suzuki K., Rikiyama T. (2024). New Cutoff of CA19-9 Values for Predicting Pancreatic Cancer Using Liquid Biopsy. J. Clin. Oncol..

[79] van Manen L., Groen J.V., Putter H., Vahrmeijer A.L., Swijnenburg R.-J., Bonsing B.A., Mieog J.S.D. (2020). Elevated CEA and CA19-9 Serum Levels Independently Predict Advanced Pancreatic Cancer at Diagnosis. Biomarkers.

[80] Poruk K.E., Gay D.Z., Brown K., Mulvihill J.D., Boucher K.M., Scaife C.L., Firpo M.A., Mulvihill S.J. (2013). The Clinical Utility of CA 19-9 in Pancreatic Adenocarcinoma: Diagnostic and Prognostic Updates. Curr. Mol. Med..

[81] Cai M., He H., Hong S., Weng J. (2023). Synergistic Diagnostic Value of Circulating Tumor Cells and Tumor Markers CEA/CA19-9 in Colorectal Cancer. Scand. J. Gastroenterol..

[82] He C.-Z., Zhang K.-H., Li Q., Liu X.-H., Hong Y., Lv N.-H. (2013). Combined Use of AFP, CEA, CA125 and CAl9-9 Improves the Sensitivity for the Diagnosis of Gastric Cancer. BMC Gastroenterol..

[83] Koopmann J., Rosenzweig C.N.W., Zhang Z., Canto M.I., Brown D.A., Hunter M., Yeo C., Chan D.W., Breit S.N., Goggins M. (2006). Serum Markers in Patients with Resectable Pancreatic Adenocarcinoma: Macrophage Inhibitory Cytokine 1 versus CA19-9. Clin. Cancer Res..

[84] Koopmann J., Buckhaults P., Brown D.A., Zahurak M.L., Sato N., Fukushima N., Sokoll L.J., Chan D.W., Yeo C.J., Hruban R.H. (2004). Serum Macrophage Inhibitory Cytokine 1 as a Marker of Pancreatic and Other Periampullary Cancers. Clin. Cancer Res..

[85] Capello M., Bantis L.E., Scelo G., Zhao Y., Li P., Dhillon D.S., Patel N.J., Kundnani D.L., Wang H., Abbruzzese J.L. (2017). Sequential Validation of Blood-Based Protein Biomarker Candidates for Early-Stage Pancreatic Cancer. J. Natl. Cancer Inst..

[86] Yoneyama T., Ohtsuki S., Honda K., Kobayashi M., Iwasaki M., Uchida Y., Okusaka T., Nakamori S., Shimahara M., Ueno T. (2016). Identification of IGFBP2 and IGFBP3 As Compensatory Biomarkers for CA19-9 in Early-Stage Pancreatic Cancer Using a Combination of Antibody-Based and LC-MS/MS-Based Proteomics. PLoS ONE.

[87] Boyd L.N.C., Ali M., Comandatore A., Garajova I., Kam L., Puik J.R., Fraga Rodrigues S.M., Meijer L.L., Le Large T.Y.S., Besselink M.G. (2023). Prediction Model for Early-Stage Pancreatic Cancer Using Routinely Measured Blood Biomarkers. JAMA Netw. Open.

[88] Perets R., Greenberg O., Shentzer T., Semenisty V., Epelbaum R., Bick T., Sarji S., BenIzhak O., Sabo E., Hershkovitz D. (2018). Mutant KRAS Circulating Tumor DNA Is an Accurate Tool for Pancreatic Cancer Monitoring. Oncologist.

[89] Pu X., Zhang C., Ding G., Gu H., Lv Y., Shen T., Pang T., Cao L., Jia S. (2024). Diagnostic Plasma Small Extracellular Vesicles miRNA Signatures for Pancreatic Cancer Using Machine Learning Methods. Transl. Oncol..

[90] Mayerle J., Kalthoff H., Reszka R., Kamlage B., Peter E., Schniewind B., Maldonado S.G., Pilarsky C., Heidecke C.-D., Schatz P. (2018). Metabolic Biomarker Signature to Differentiate Pancreatic Ductal Adenocarcinoma from Chronic Pancreatitis. Gut.

[91] Yang Z., LaRiviere M.J., Ko J., Till J.E., Christensen T., Yee S.S., Black T.A., Tien K., Lin A., Shen H. (2020). A Multianalyte Panel Consisting of Extracellular Vesicle miRNAs and mRNAs, cfDNA, and CA19-9 Shows Utility for Diagnosis and Staging of Pancreatic Ductal Adenocarcinoma. Clin. Cancer Res..

[92] García-Ortiz M.V., Cano-Ramírez P., Toledano-Fonseca M., Cano M.T., Inga-Saavedra E., Rodríguez-Alonso R.M., Guil-Luna S., Gómez-España M.A., Rodríguez-Ariza A., Aranda E. (2023). Circulating NPTX2 Methylation as a Non-Invasive Biomarker for Prognosis and Monitoring of Metastatic Pancreatic Cancer. Clin. Epigenet..

[93] Yeh C., Zhou M., Sigel K., Jameson G., White R., Safyan R., Saenger Y., Hecht E., Chabot J., Schreibman S. (2023). Tumor Growth Rate Informs Treatment Efficacy in Metastatic Pancreatic Adenocarcinoma: Application of a Growth and Regression Model to Pivotal Trial and Real-World Data. Oncologist.

[94] Vasconcelos A.O., Vieira L.M., Rocha C.R.C., Beltrão E.I.C. (2024). The Contribution of Fucosyltransferases to Cancer Biology. Braz. J. Biol..

[95] Guo M., Luo G., Lu R., Shi W., Cheng H., Lu Y., Jin K., Yang C., Wang Z., Long J. (2017). Distribution of Lewis and Secretor Polymorphisms and Corresponding CA19-9 Antigen Expression in a Chinese Population. FEBS Open Bio.

[96] Abe T., Koi C., Kohi S., Song K.-B., Tamura K., Macgregor-Das A., Kitaoka N., Chuidian M., Ford M., Dbouk M. (2020). Gene Variants That Affect Levels of Circulating Tumor Markers Increase Identification of Patients With Pancreatic Cancer. Clin. Gastroenterol. Hepatol..

[97] Ando Y., Goggins M. (2024). ASO Author Reflections: Using a CA19-9 Tumor Marker Gene Test to Assess Outcome after Pancreatic Cancer Surgery. Ann. Surg. Oncol..

[98] Dbouk M., Abe T., Koi C., Ando Y., Saba H., Abou Diwan E., MacGregor-Das A., Blackford A.L., Mocci E., Beierl K. (2023). Diagnostic Performance of a Tumor Marker Gene Test to Personalize Serum CA19–9 Reference Ranges. Clin. Cancer Res..

[99] Ghosh I., Bhattacharjee D., Das A.K., Chakrabarti G., Dasgupta A., Dey S.K. (2013). Diagnostic Role of Tumour Markers CEA, CA15-3, CA19-9 and CA125 in Lung Cancer. Indian J. Clin. Biochem..

[100] Kothari A., Thalji S., Aldakkak M., SenthilKumar G., Belbahri M., Shaik T., Jaraczewski T., Merrill J., Ramamurthi A., Banerjee A. (2025). AI-Derived Electronic Tumor Marker (E19-9) Can Measure Treatment Response and Outcomes in CA19-9 Non-Producers with Pancreatic Ductal Adenocarcinoma. Res. Sq..

[101] MorrisStiff G., Taylor M. (2012). Ca19-9 and Pancreatic Cancer: Is It Really That Good?. J. Gastrointest. Oncol..

[102] Erden G., Barazi A.O., Tezcan G., Yildirimkaya M.M. (2008). Biological Variation and Reference Change Values of CA 19-9, CEA, AFP in Serum of Healthy Individuals. Scand. J. Clin. Lab. Investig..

[103] Li W., Sun G., Zhu R., Li P., Wang L. (2025). Case Report: Sequential PTCD and Biliary Seed Stent Combined with Targeted-Immunotherapy for Advanced Pancreatic Cancer with Malignant Obstructive Jaundice: A Multidisciplinary Approach. Front. Oncol..

[104] Lyu S., Wang F., Ren Z., He Q. (2021). Correlation Analysis of CA19-9/Glutamyltransferase Ratio and Long-Term Survival for Patients with Pancreatic Head Cancer under Different Bilirubin Levels. Asian J. Surg..

